# The non-synonymous SNP, R1150W, in *SCN9A* is not associated with chronic widespread pain susceptibility

**DOI:** 10.1186/1744-8069-8-72

**Published:** 2012-09-24

**Authors:** Kate L Holliday, Wendy Thomson, Tuhina Neogi, David T Felson, Ke Wang, Frederick C Wu, Ilpo T Huhtaniemi, Gyorgy Bartfai, Felipe Casanueva, Gianni Forti, Krzysztof Kula, Margus Punab, Dirk Vanderschueren, Gary J Macfarlane, Michael A Horan, William Ollier, Antony Payton, Neil Pendleton, John McBeth

**Affiliations:** 1 Arthritis Research UK Epidemiology Unit, University of Manchester, Manchester Academic Health Science Centre, Manchester, UK; 2 Section of Clinical Epidemiology Research and Training Unit, Boston University School of Medicine, Boston, Massachusetts, USA; 3 Andrology Research Unit, Developmental & Regenerative Biomedicine Research Group, University of Manchester, Manchester, UK; 4 Department of Surgery and Cancer, Imperial College London, London, UK; 5 Department of Obstetrics, Gynaecology and Andrology, Albert Szent-Gyorgy Medical University, Szeged, Hungary; 6 Department of Medicine, Santiago de Compostela University, Complejo Hospitalario Universitario de Santiago (CHUS), CIBER de Fisiopatología Obesidad y Nutricion (CB06/03), Instituto Salud Carlos III, Santiago de Compostela, Spain; 7 Andrology Unit, Department of Clinical Physiopathology, University of Florence, Florence, Italy; 8 Department of Andrology and Reproductive Endocrinology, Medical University of Lodz, Lodz, Poland; 9 Andrology Unit, United Laboratories of Tartu University Clinics, Tartu, Estonia; 10 Department of Andrology and Endocrinology, Katholieke Universiteit Leuven, Leuven, Belgium; 11 Aberdeen Pain Research Collaboration (Epidemiology Group), University of Aberdeen, Aberdeen, UK; 12 Clinical Gerontology, Hope Hospital, University of Manchester, Manchester, UK; 13 Centre for Integrated Genomic Medical Research, University of Manchester, Manchester, UK

**Keywords:** Chronic widespread pain, Single nucleotide polymorphism, Voltage-gated sodium channel, Population-based cohorts

## Abstract

**Background:**

Mutations in *SCN9A,* encoding the alpha subunit of the voltage-gated sodium channel (Na_v_1.7), have caused severe pain disorders and congenital insensitivity to pain. The aim of this study was to validate the previously reported association between a common non-synonymous polymorphism (R1150W, rs6746030) in *SCN9A* and chronic widespread pain (CWP), in independent population-based cohorts.

**Findings:**

Genotype data for rs6746030 was available in four population-based cohorts (EPIFUND, the European Male Ageing Study (EMAS), the Framingham study and the Dyne Steel DNA Bank of Ageing and Cognition). Pain was assessed using body manikins and CWP was scored using American College of Rheumatology (ACR) criteria in all cohorts, except the Framingham study which assessed widespread pain (WP) using ACR criteria on a joint pain homunculus. Controls were subjects who reported no pain. Logistic regression (additive genetic model) was used to test for association between rs6746030 and CWP compared to controls, adjusting for study centre in EMAS. Generalised estimating equation regression was used to test for association between rs6746030 and WP, whilst accounting for relatedness between subjects in the Framingham study.

Genotype data for rs6746030 was available for 1071 CWP cases and 3212 controls. There was no significant association between CWP and rs6476030 in individual cohorts or when combined in a fixed-effects meta-analysis (Odds Ratio = 0.96 (95% confidence interval 0.82, 1.11) p = 0.567).

**Conclusions:**

In contrast to a previous study, no association between a non-synonymous polymorphism in *SCN9A* and CWP was observed in multiple population-based cohorts.

## Introduction

*SCN9A* encodes an alpha subunit of the voltage-gated sodium channel Na_v_1.7, which is expressed primarily in nociceptive dorsal root ganglia (DRG) neurons and is involved in the production of action potentials. Gain-of-function mutations in *SCN9A* have caused a number of severe pain syndromes through increased activity of the channel and loss-of-function mutations in *SCN9A* have caused congenital insensitivity to pain [1].

Many complex trait susceptibility genes contain mutations causing severe monogenic disorders with phenotypic similarity in families. Consequently, it has been hypothesised that common variation in *SCN9A* may influence pain perception and susceptibility to chronic pain disorders. A non-synonymous single nucleotide polymorphism (SNP), rs6746030G → A, in *SCN9A* which causes an arginine(R) to tryptophan(W) substitution, was recently associated with increased pain scores in subjects with clinical pains (including Osteoarthritis (OA)), increased sensitivity to experimental pain in healthy individuals and a non-significant increase in post-operative pain scores [2]. The 1150 W allele has been shown to depolarise activation and increase the firing rate in DRG neurons in response to depolarisation [3] and steeper voltage dependence for slow inactivation for 1150 W compared to 1150R has been reported suggesting that the variant allele may make neurons more sensitive to painful stimuli [2].

Valdes et al. (2011) failed to validate the association between rs6746030 and pain scores in OA, reported by Reimann et al. (2010), despite a larger study sample. They did, however, report an increased odds of reporting widespread pain (WP) with the A allele [4]. The aim of this study was to validate the Valdes et al. (2011) finding to determine if a putative functional polymorphism, rs6746030, in *SCN9A* is associated with CWP.

## Methods

Data was utilised from four population-based cohorts. The Framingham study is a longitudinal multi-generational study conducted in Framingham, Massachusetts, USA since 1948. Pain data was collected at exam 22 in the original cohort and at exams 5–7 for the offspring cohort. In the European Male Ageing Study (EMAS), men aged 40–79 years were recruited from population registers in 8 European centres [5]. Ethical approval for the study was obtained at the co-ordinating centre and each participating centre. [6]. In the EPIFUND cohort, subjects aged 25–65 years were recruited via three primary care practices in North-West England. Ethical approval was obtained from Manchester local research ethics commitee. The Dyne Steele DNA bank for ageing and cognition (DSDBAC) includes elderly individuals participating in a longitudinal study of cognition recruited via advertisements in Manchester and Newcastle, UK, in 1983 [6]. Pain questionnaires were sent to subjects remaining in the cohort in 2007 (aged 67–96 years). Ethical approval was obtained from the University of Manchester. All participants gave informed consent.

Pain status was assessed using a questionnaire and joint pain homunculus in the Framingham Study. Subjects were scored as having WP if they reported pain in joints in contra-lateral body quadrants, above and below the waist and in the axial skeleton in accordance with American College of Rheumatology (ACR) criteria. In the other cohorts, pain was assessed using a questionnaire and body manikins on which subjects shaded the site of any pain and chronic widespread pain (CWP), WP present for at least 3 months, was scored using ACR criteria. Controls were subjects who reported no pain.

In silico genotype data for rs6746030 was utilised for the Framingham and DSDBAC cohorts. The Framingham SNP Health Association Resource (SHARe) project genotyped 9274 participants using the Affymetrix 500 K mapping array and the Affymetrix 50 K supplemental arrays, 3850 of which met sample quality controls requirements (individuals were excluded if they were genotyped for < 95% of SNPs, had excess heterozygosity or >100 mendelian errors) and had pain data available. DSDBAC was genotyped using the Illumina610-Quadv1 chip. 602 subjects passed sample QC (individuals were excluded if they were genotyped for <95% of SNPs, for gender discrepancy, relatedness (Identitiy By Descent (IBD) sharing >0.25) and evidence of non-Caucasian ancestry (by multi-dimensional scaling)) and had pain data available. Rs6746030 was genotyped using the Sequenom iPLEX Gold massARRAY platform (Sequenom Inc, San Diego, USA) following the manufacturer’s instructions in EPIFUND and EMAS.

Generalised estimating equation regression was used to test for association between rs6746030 and WP in the Framingham study under an additive genetic model, accounting for relatedness between subjects and using genomic control to adjust for population stratification in R. Logistic regression under an additive genetic model was used to test for association between rs6746030 and CWP in the other cohorts in PLINK [7] adjusting for study centre in EMAS. Effect estimates were combined in meta-analysis using STATA v10.

## Results

In silico genotype data for rs6746030, met SNP quality control criteria in the Framingham (genotyped in ≥97% of subjects, Hardy-Weinberg Equilibrium (HWE) p > 1 x10^-6^) and DSDBAC (genotyped in ≥98% of subjects, HWE p > 1 x10^-3^) cohorts. Rs6746030 was genotyped in EPIFUND and EMAS in 100% of subjects with HWE p > 0.05. The characteristics of the cases (n = 1071) and controls (n = 3214) in the four cohorts are shown in Table 1. Prevalence of CWP and being pain-free varied between cohorts in accordance with age, gender and study design.

**Table 1:** Characteristics of the study populations

**Cohort**	**Framingham**	**EMAS**	**EPIFUND**	**DSDBAC**
Country	USA	Pan-European	UK	UK
Pain assessment	Homunculus	Manikin	Manikin	Manikin
Cases^a^				
N subjects (%)	572 (15)	208 (8)	197 (18)	94 (16)
Age, years mean (SD)	60.4 (11.4)	60.5 (10.9)	51.1 (9.0)	79.6 (4.7)
Gender (% female)	M/F (67)	M	M/F (72)	M/F (86)
Controls^b^				
N subjects (%)	1587 (41)	973 (39)	395 (35)	259 (43)
Age, years mean (SD)	57.9 (13.1)	59.7 (10.9)	48.2 (10.3)	80.5 (5.7)
Gender (% female)	M/F (51)	M	M/F (55)	M/F (71)

^a^ cases are subjects with chronic widespread pain (ACR criteria) except Framingham which is widespread pain; ^b^ controls are pain-free subjects.

**Figure 1:**
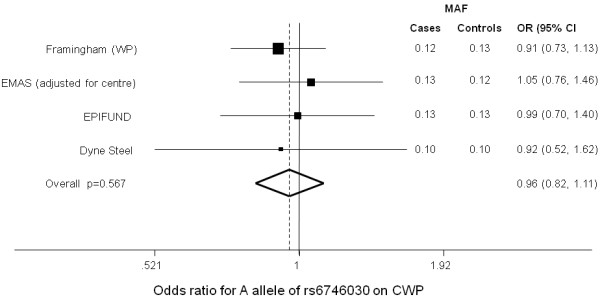
**Forest plot for the odds ratio (95%CI) of the A allele of rs6746030 on CWP by cohort and combined in a meta-analysis (fixed-effects).** Odds ratios () and their 95% confidence intervals (CI) are shown for the effect in each cohort. The odds ratio for all cohorts combined (in a fixed effects meta-analysis) is shown by the dashed line, with the extremes of the diamond shape representing confidence intervals. The line at 1 represents the null. If the 95%CI crosses this line then there is no association.

With 1-sided 5% type I error and a minor allele frequency (MAF) of 13%, this study had 80% power to detect an odds ratio of 1.20 for CWP compared to pain-free controls and 99% power to detect an odds ratio of 1.40 as observed by Valdes et al. (2011), calculated using Quanto [8]. There was a non-significant decrease in the prevalence of the A allele in subjects with WP and CWP in the Framingham and DSDBAC cohorts, respectively. There was a non-significant increase in prevalence of the A allele in CWP cases in EMAS, which did not significantly differ between study centres (p = 0.30), and no difference in prevalence of the A allele between cases and controls in EPIFUND. There was no evidence of heterogeneity (I^2^ = 0%, p = 0.891) between cohorts, therefore a fixed-effects meta-analysis was used to combine effect estimates, which resulted in an odds ratio = 0.96 (95% confidence interval 0.82, 1.11) p = 0.567 per A allele of rs6746030 on CWP (Figure 1).

## Discussion

This study attempted to validate an association between rs6746030 in the *SCN9A* gene and reporting pain at multiple body sites in four independent cohorts. No evidence of association with CWP was observed, conflicting with a previous report that the minor (A) allele was associated with increased odds of reporting pain in multiple sites [4].

The findings of this study and the Valdes et al. study are equivocal; this may be due to a number of factors. Firstly, the criteria used to classify cases with widespread pain and controls differed between studies. The majority (76.1%) of subjects in the Valdes et al. study were recruited as part of case–control study of OA with the remainder recruited from two population based studies. Subjects were classified as having multiple regional pain using different definitions in each sub-study, however, the authors reported no between-study heterogeneity. Secondly, the prevalence of WP/CWP reported here, 8-18% was higher than in Valdes et al. (2011), 4-9%. It is possible that the case phenotype in the present study was more heterogeneous and/or includes subjects with less severe or disabling pain and rs6746030 may associate with severe/disabling widespread pain. Thirdly, in the present study, controls were subjects reporting no pain to eliminate the possibility of a false negative association occurring due to the presence of regional pain in the control group. However, in the Valdes et al. study control subjects may or may not have had pain suggesting that the association was specifically with multi-site pain rather than pain *per se*. Finally, the original observation was adjusted for age, sex and body mass index. Individuals with widespread pain have increased rates of other painful disorders such as irritable bowel syndrome and chronic oro-facial pain and the results from the Valdes et al. (2011) study may reflect a higher “pain-load” that was not detected in this study due to phenotype heterogeneity.

The limitations of this study were that the pain assessment and definition differed between cohorts. A joint pain assessment, which did not include chronicity, was used for the Framingham study. Consequently, cases in the Framingham cohort may represent a different group to the other cohorts; however, the majority of chronic pain reported is at joint sites and widespread pain is usually chronic. In addition, the controls in the Framingham cohort may have had some pain at non-joint sites and may not represent a truly pain-free group. The cohorts also differed in age, gender, geographic location and individual cohorts lacked power to detect an association. No data on ethnicity was available for the EPIFUND cohort, however, the majority are thought to be white British [9] and only information on self-reported ancestry was available in EMAS.

CWP will resolve in some individuals and persists in others. Genetic factors may play a more important role in persistent and/or disabling CWP; although, this has yet to be demonstrated. If so, comparing individuals with persistent and/or disabling CWP to pain-free controls may be a more powerful approach to identifying susceptibility loci for chronic pain. Given that pain was assessed at a single time-point in the majority of the cohorts utilised in this study and associated disability data was not available, we were unable to test this in the current study; however, this approach could be fruitful if adopted in future studies.

Our findings also conflict with those of Reimann et al. (2010), who observed that the A allele of rs6746030 was associated with reporting higher pain scores in subjects with a variety of clinical pains and increased pain sensitivity in healthy individuals [2]. This may suggest that the SNP is associated with the severity of pain reported within a pain state rather than the presence of widespread pain, as tested here. However, Valdes et al. (2011) did not observe an association between rs6746030 and pain scores in knee OA in over 1000 subjects [4].

The lack of association reported here, despite the large sample size (1071 cases and 3214 controls), suggests that this SNP is not a susceptibility marker for CWP. Other SNPs within the gene or in regulatory regions for *SCN9A* may be important in CWP susceptibility and may influence the effect of rs6746030 but have yet to be investigated. Environmental factors, not controlled for in the present study could also potentially mask a true effect.

In conclusion, we find no evidence of association for rs6746030 in *SCN9A*, previously shown to play a role in pain perception, with CWP in a meta-analysis of four population-based cohorts. However, *SCN9A* remains a strong candidate gene for chronic pain and is worthy of investigation in large-scale genetic association studies. Harmonisation of pain phenotypes to allow cohorts to be combined is required to facilitate such studies and to elucidate the genetic risk factors for chronic pain.

## Competing interests

The authors have no competing interests to declare.

## Authors’ contributions

KLH designed the study, analysed the data, interpreted the findings and drafted the manuscript. WT & JM contributed to the design of the study, acquisition of data and interpretation of the findings. KW contributed to the analysis of the data. TN, DTF, FCW, ITH, GB, FC, GF, KK, MP, DV, GJM, MAH, WO, AP and NP contributed to the acquisition of data. All authors critically reviewed the manuscript and approved the final version for publication.

## References

[B1] Drenth JP Waxman SG Mutations in sodium-channel gene SCN9A cause a spectrum of human genetic pain disorders J Clin Invest 2007 117 3603 3609 10.1172/JCI33297 18060017 PMC2096434

[B2] Reimann F Cox JJ Belfer I Diatchenko L Zaykin DV McHale DP Drenth JP Dai F Wheeler J Sanders F Pain perception is altered by a nucleotide polymorphism in SCN9A Proc Natl Acad Sci U S A 2010 107 5148 5153 10.1073/pnas.0913181107 20212137 PMC2841869

[B3] Estacion M Harty TP Choi JS Tyrrell L Dib-Hajj SD Waxman SG A sodium channel gene SCN9A polymorphism that increases nociceptor excitability Ann Neurol 2009 66 862 866 10.1002/ana.21895 20033988

[B4] Valdes AM Arden NK Vaughn FL Doherty SA Leaverton PE Zhang W Muir KR Rampersaud E Dennison EM Edwards MH Role of the Na(V)1.7 R1150W amino acid change in susceptibility to symptomatic knee osteoarthritis and multiple regional pain Arthritis Care Res 2011 63 440 444 10.1002/acr.20375 21031562

[B5] Lee D O'Neill T Pye S Silman A Finn J Pendleton N Tajar A Bartfai G Casanueva F Forti G The European Male Ageing Study (EMAS): design, methods and recruitment Int J Androl 2009 32 11 24 10.1111/j.1365-2605.2008.00879.x 18328041

[B6] Rabbitt PM McInnes L Holland F Bent N Abson V Pendleton N Horan M The University of Manchester Longitudinal Stusy of Cognotion in Normal Helaty Old Age, 1983 through 2003 Aging Neuropsychol Cognit 2004 11 245 279 10.1080/13825580490511116

[B7] Purcell S Neale B Todd-Brown K Thomas L Ferreira MA Bender D Maller J Sklar P de Bakker PI Daly MJ PLINK: a tool set for whole-genome association and population-based linkage analyses Am J Hum Genet 2007 81 559 575 10.1086/519795 17701901 PMC1950838

[B8] Gauderman WJ Morrison JM QUANTO 1.1: A computer program for power and sample size calculations for genetic-epidemiology studies 2006

[B9] Office for National Statistics 2001 Census: Standard Area Statistics (England and Wales) 2001

